# Responses of soil hydrolytic enzymes, ammonia-oxidizing bacteria and archaea to nitrogen applications in a temperate grassland in Inner Mongolia

**DOI:** 10.1038/srep32791

**Published:** 2016-09-06

**Authors:** Xinyu Zhang, Yuqian Tang, Yao Shi, Nianpeng He, Xuefa Wen, Qiang Yu, Chunyu Zheng, Xiaomin Sun, Weiwen Qiu

**Affiliations:** 1 Key Laboratory of Ecosystem Network Observation and Modeling, Institute of Geographic Sciences and Natural Resources Research, Chinese Academy of Sciences, Beijing 100101, China; 2 School of Geographic Sciences, Northeast Normal University, Changchun 130024, China; 3 State Key Laboratory of Forest and Soil Ecology, Institute of Applied Ecology, Chinese Academy of Sciences, Shenyang 110016, China; 4 The New Zealand Institute for Plant and Food Research Limited, Private Bag 4704, Christchurch, New Zealand

## Abstract

We used a seven-year urea gradient applied field experiment to investigate the effects of nitrogen (N) applications on soil N hydrolytic enzyme activity and ammonia-oxidizing microbial abundance in a typical steppe ecosystem in Inner Mongolia. The results showed that N additions inhibited the soil N-related hydrolytic enzyme activities, especially in 392 kg N ha^−1 ^yr^−1^ treatment. As N additions increased, the *amoA* gene copy ratios of ammonia-oxidizing archaea (AOA) to ammonia-oxidizing bacteria (AOB) decreased from 1.13 to 0.65. Pearson correlation analysis showed that the AOA gene copies were negatively related with NH_4_^+^-N content. However, the AOB gene copies were positively correlated with NO_3_^−^-N content. Moderate N application rates (56–224 kg N ha^−1 ^yr^−1^) accompanied by P additions are beneficial to maintaining the abundance of AOB, as opposed to the inhibition of highest N application rate (392 kg N ha^−1 ^yr^−1^) on the abundance of AOB. This study suggests that the abundance of AOB and AOA would not decrease unless N applications exceed 224 kg N ha^−1 ^yr^−1^ in temperate grasslands in Inner Mongolia.

Soil fertility decline is considered as a primary cause for low productivity of grasslands in northern China (approximately 150 million ha)^1^. Nitrogen (N) has been identified as the main nutrient that limits biomass and above-ground net primary production^2^. Therefore, grassland ecosystems are likely to be highly sensitive to N additions, and N may improve plant productivity and soil organic carbon stocks in this area^1^,^3^. N fertilization practices can also markedly suppress community species richness, and community stability of grassland ecosystems in N-limited environments^4^,^5^,^6^. Excess N has been widely recognized as one of major drivers of biodiversity loss in agro-ecosystems. Bai *et al*.^3^ reported that N-induced species loss occurred in mature Eurasian grasslands when N applications exceeded 17.5 kg ha^−1 ^yr^−1^. Changes in above-ground biomass and species richness were observed in both mature and degraded ecosystems when N applications exceeded 105 kg ha^−1 ^yr^−1^. It is important to understand the response of nitrification to N applications so as to predict the potentials of nitrate leaching and nitrous oxide (N_2_O) emissions. In addition, effects of enhanced N additions on the activities of soil enzymes and microbial communities that are involved in N transformations need to be understood. However, to date, there is no information about the N loading thresholds of the important functional microbes that mediate N transformations (e.g., ammonia oxidizers) below-ground in this area.

Microbial N acquisition activity can be highly sensitive to N additions to grassland^7^,^8^. Activities of β-1, 4-N-acetylglucosaminidase (NAG) and leucine aminopeptidase (LAP) control the release of plant available N from organic compounds^9^. The responses of soil enzyme activity to nitrogen fertilization in arid soils are distinct from those in other soils. Wang *et al*.^10^ suggested that β-glucosidase and acid phosphomonoesterase in temperate grasslands did not change when N was applied, whereas Zhou *et al*.^11^ found that N additions over just a two year period in the Gutbantunggut Desert led to significant decreases in urease activity. To date, there is little information about how NAG and LAP activities respond to N application in steppe ecosystems, and how these activities relate to soil N availability over the long term.

Ammonia oxidation is thought to be the primary step of nitrification, in which ammonia oxidizers are responsible to limit the subsequent nitrification rate, regulating the balance between ammonium, nitrate, and nitrite. So this process can affect soil nitrogen availability; and is therefore vital to the nitrogen cycle in terrestrial ecosystems^12^. The ammonia monooxygenase (AMO), which is a periplasm-associated enzyme, catalyzes the reaction of oxidizing ammonia to hydroxylamine in the first step of nitrification^13^. Ammonia-oxidizing archaea (AOA) and ammonia-oxidizing bacteria (AOB), which have the ammonia monooxygenase alpha subunit gene (*amoA*), are thought to play important roles in catalyzing this step^14^,^15^. However, due to the differences between AOA and AOB in cellular, genomic and physiological levels, AOA is more energy-effective^16^ and has relatively higher affinity to ammonia than AOB^17^. As reported, AOA prefer ammonia-poor and acidic conditions; while AOB favor N-rich and alkaline environments^18^. Given the background of N-limit in the grassland in Inner Mongolia, it is necessary to assess the competition mechanism of AOA and AOB in ammonia oxidation. Previous studies have examined the effects of N additions on the abundance of AOA and AOB in grassland ecosystems^13^,^19^,^20^. Results showed that urea-N additions of up to 150 kg N ha^−1 ^yr^−1^ led to significant increases in AOB, but not in AOA, which suggested that AOB was more sensitive to N addition than AOA^19^. Nitrogen enrichment may cause phosphorus (P) being the main limiting nutrient and P fertilization could influence AOA and AOB abundance^20^. Phosphorus limitation could change the effect of N application on AOA and AOB, however, it is not clear how N application rates influence AOA and AOB abundance in semiarid temperate grasslands in the presence of P.

In view of the central role played by soil microorganisms in soil N cycling, it is therefore necessary to determine the effects and thresholds of long-term N applications on soil microbial activity and N cycling biochemical processes in grassland ecosystems. In this study, we used a seven-year field experiment that comprised five levels of urea gradient additions combined with P addition in the Xilinguole grassland area, Inner Mongolia. The main objectives were to (1) explore the effects of N applications on soil N hydrolytic enzyme activities; (2) quantify the abundance of ammonia-oxidizing archaea (AOA) and ammonia-oxidizing bacteria (AOB) at different N application levels without limiting P; and (3) find a threshold for N applications that the enzyme activities, AOA and AOB will be changed if the N application is above the threshold.

## Results

### Soil physico-chemical properties and net nitrification rates

**Figure 1:**
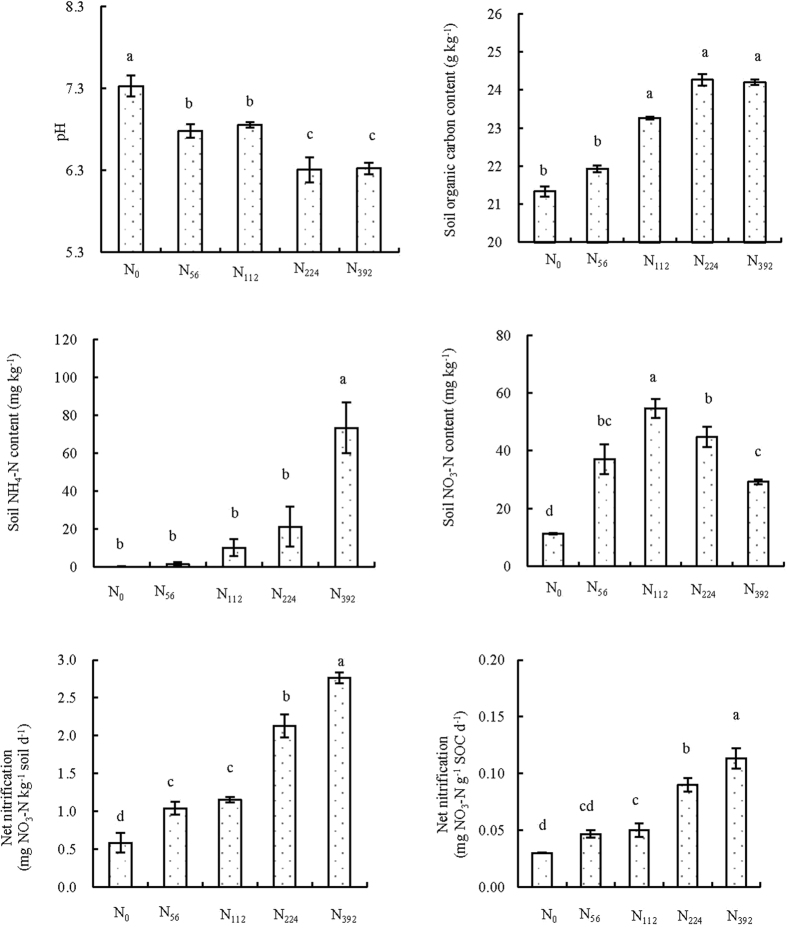
Soil physico-chemical properties and net nitrification under different N addition treatments (mean ± standard error, n = 3). Bars labeled with different letters were significantly different (P < 0.05). The same labelling applies for other figures.

Nitrogen additions resulted in significant decreases in soil pH, and increased soil organic carbon (SOC) and inorganic N contents (Fig. 1). Soil pH decreased from 7.3 to 6.3 due to the N additions. SOC content increased from 21.6 to 24.3 g kg^−1^, with significant difference (P < 0.05) between the treatments of below and above 112 kg ha^−1^ N application. NH_4_^+^-N and NO_3_^−^-N were greater in the N treatments than in the control treatment (N_0_). Maximum NH_4_-N level was observed in the treatment with the highest rate of applied fertilizer, whereas the NO_3_-N level peaked at the medial N amount adding treatment of 112 kg ha^−1^ rate. The net nitrification rate increased from 0.6 to 2.8 mg NO_3_^−^-N kg^−1^ soil d^−1^ after N additions, i.e. from 0.03 to 0.11 mg NO_3_^−^-N g^−1^ SOC d^−1^ after N additions, with significant increases of net nitrification adjusted to SOC observed in all the N addition treatments.

### Soil N related hydrolytic enzyme activities

**Figure 2:**
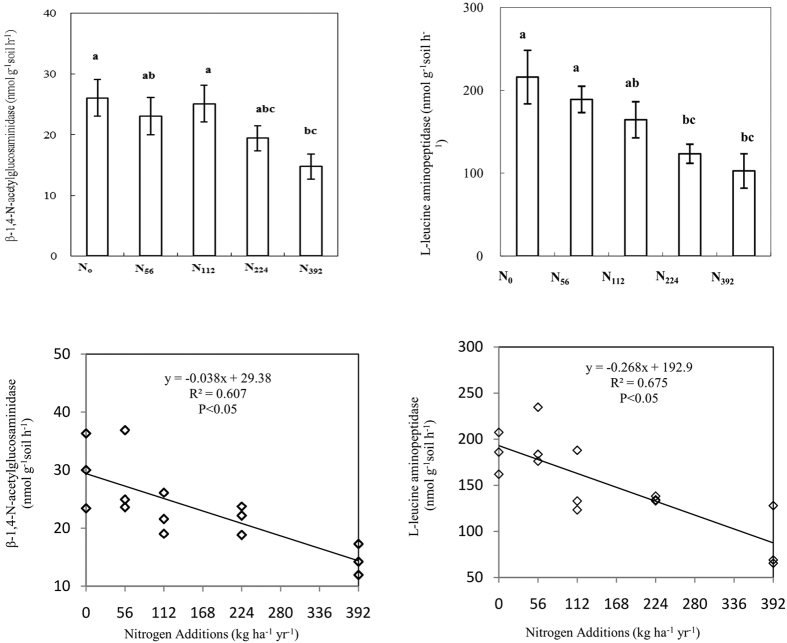
Effects of N additions on soil β-1,4-N-acetylglucosaminidase (NAG) and L-leucine aminopeptidase (LAP) activities, and regression analysis between soil nitrogen related hydrolase activities and the amount of nitrogen additions. (mean ± standard error, n = 3).

Nitrogen-related hydrolytic enzyme activities decreased as a result of high N application rates (Fig. 2). The NAG enzyme activities decreased by between 4–42% in response to N additions, and a significant decrease was observed for the N_392_ treatment (Fig. 2). The LAP enzyme activities decreased by between 13–52% owing to the N additions, and significant decreases were observed for the N_224_ and N_392_ treatments (Fig. 2). Regression analysis showed that both LAP and NAG activities were decreased linearly with the amount of N additions (Fig. 2, P < 0.05). Both LAP and NAG activities were positively related with pH, and negatively correlated with NH_4_^+^-N, SOC content and nitrification rates (Table 1). There were no significant correlations between soil NO_3_^−^-N content and NAG and LAP activities (Table 1).

### Ammonia-oxidizing archaea and bacteria

**Figure 3:**
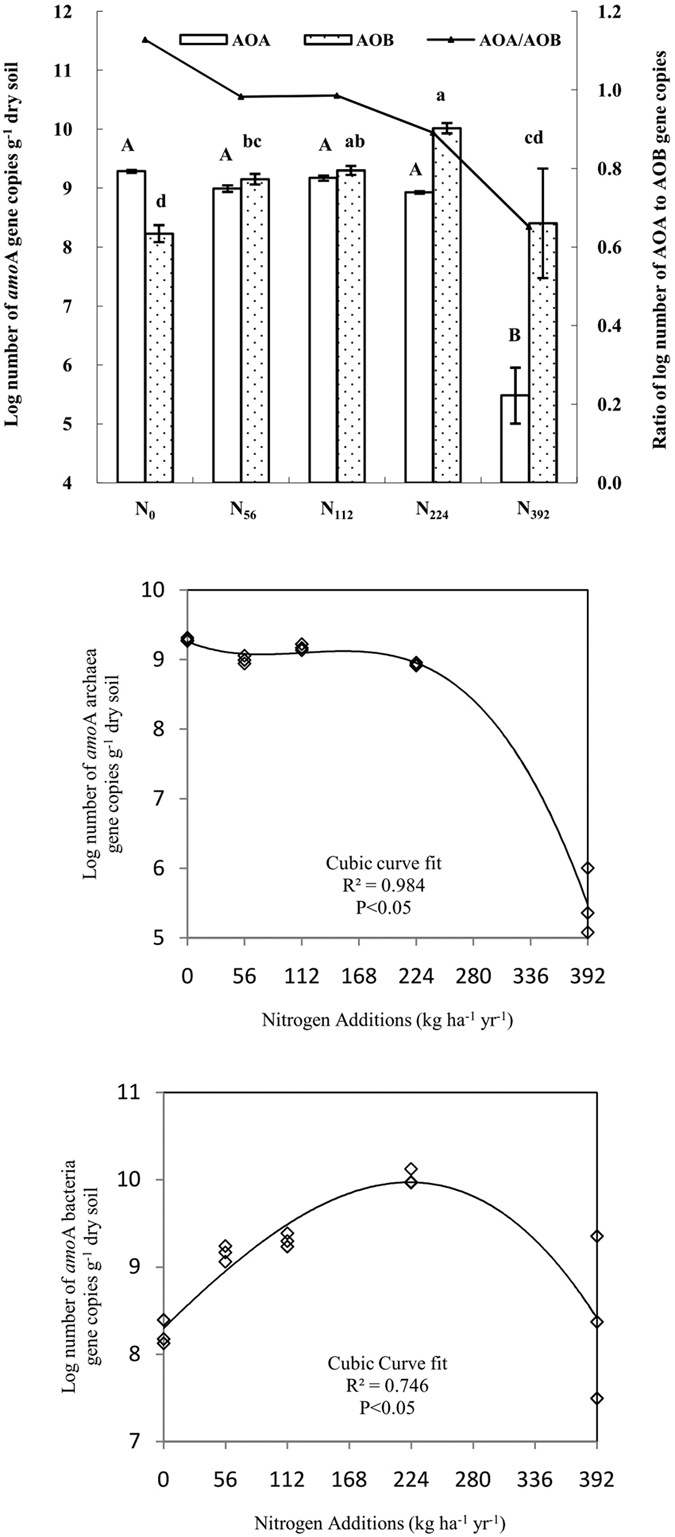
Effects of N additions on soil ammonia-oxidizing archaea and bacteria gene copies, and regression analysis between soil ammonia-oxidizing archaea, bacteria gene copies and the amount of nitrogen additions. (mean ± standard error, n = 3).

Gene copy numbers were used to estimate AOB and AOA abundances. The AOB gene copy numbers ranged from 1.45 × 10^9^ to 1.06 × 10^10 ^g^−1^ soil, and increased significantly in the N_56_, N_112_ and N_224_ treatments relative to the N_0_ treatment (Fig. 3). However, AOA gene copy numbers in the N_56_, N_112_ and N_224_ treatments, ranging from 8.52 × 10^8^ to 1.5 × 10^9 ^g^−1^ soil, were not significantly different from the control. The AOA gene copy numbers were lower in the N_392_ treatment than in all other treatments. The ratio of AOA to AOB gene copy numbers decreased from 1.13 in N_0_ to 0.65 in N_392_ (Fig. 3). Regression analysis showed that there was a non-linear (cubic) relationship between ammonia-oxidizing archaea and bacteria gene copies and the N application rate (Fig. 3, P < 0.05). The N application threshold beyond which the abundance of soil ammonia-oxidizing archaea and bacteria declined was 224 kg N ha^−1 ^yr^−1^. Pearson correlation analysis showed that AOA gene copies were positively correlated to soil pH and negatively related to nitrification rates, NH_4_^+^-N and SOC content. Soil NO_3_^−^–N was the only soil variable with a significant relationship with AOB (Table 1).

## Discussion

### Responses of soil organic carbon to nitrogen applications

In this study, the SOC content increased significantly due to the N additions^1^ and there was a negative relationship between SOC and hydrolase. In contrast to our study, Bi *et al*.^21^ reported no significant effect of N addition on SOC content. Given the fact that their N addition amount was 100 kg N ha^−1 ^yr^−1^, which was much less than the N applied in this study, we assumed that the amount of N addition in their study was not enough to stimulate C sequestration in grassland ecosystem. The potential sequestration of C under high N additions has also been proved by He *et al*.^1^ in Inner Mongolia grassland with N additions ranging from 56 to 560 kg N ha^−1^ yr^−1^. Lu *et al*.^22^ reported minor stimulation effect of N additions on SOC content in a meta-analysis of 257 published studies. However, the number of trials in forest ecosystems selected for the meta-analysis was two-fold more than those in grassland. As a result, it may cause an underestimation of N additions on C sequestration in grassland ecosystem in the meta-analysis of Lu *et al*.^22^. Our finding is in agreement with Liu and Greaver^23^ who reported N simulated more litter input to soil in grassland than that in forest in a meta-analysis and found that N additions increased soil C storage. The increased SOC content could also be explained by the decrease of organic matter decomposition since the enzyme activity of NAG, LAP decreased with high N additions in our study.

### High N application rates inhibited the hydrolytic enzyme activities involved in N decomposition

In this long term urea gradient experiment, the response of the NAG and LAP activities was generally suppressed, especially by high N application rates. Stursova *et al*.^7^ also found that peptidase was depressed in semiarid grasslands after ten years of 10 kg N ha^−1 ^yr^−1^ NH_4_NO_3_ addition, while Saiya-Cork *et al*.^24^ found that soil LAP activity decreased dramatically in response to N additions.

Firstly, this is supported by the “microbial economics” hypothesis^25^, which predicts that production of enzymes that degraded complex substrates will decrease when assimilated resources are available, i.e. the activities of enzymes involved in the degradation of organic N compounds (e.g., protein, chitin, peptidoglycan) will decrease because of the available N enrichment. Correlation analysis suggested that NH_4_^+^-N played a major role in decreasing N-related hydrolase activity as the higher N was added (Table 1). The negative relationship between NAG and LAP and nitrification, and the absence of a relationship with NO_3_^−^-N, suggested that NO_3_^−^-N had a minimal effect on NAG and LAP (Table 1). Hydrolases are repressible enzymes, and their synthesis is down-regulated by the presence of the end product^26^,^27^, i.e. NAG and LAP synthesis are repressed by the presence of NH_4_^+^-N. The inhibition of NAG and LAP production exceeded the stimulation effects of SOC on hydrolase activities in this study.

Secondly, soil acidification can reduce microbial biomass and activities in semiarid grassland^28^,^29^. It has also been reported that soil pH is a key driver of dehydrogenase when N is added^10^. In this study, the soil pH values decreased by nearly one unit under the higher N applications (Fig. 1), both NAG and LAP activities were significantly and positively related to soil pH (Table 1). This may partly explain why NAG and LAP activities were suppressed by N additions.

Thirdly, nitrogen deposition has been widely recognized as an environmental problem that associates with biodiversity loss^3^. It was reported that there would be N-induced species loss in both mature and degraded grassland ecosystems^3^ when N applications exceeded 105 kg N ha^−1 ^yr^−1^. The decrease of the plant species richness could also contribute to the enzyme activities decline by the rhizosphere organic secretion reduction. Previous research has demonstrated that long term N additions can result in reduced soil microbial biomass as well as lower respiration rate^29^, in which factors suppressing microbial biomass may also reduce the associated enzyme activities^8^,^10^. The microbial community structure may shift in response to the urea additions^28^, and the decrease in the bacterial to fungal ratio can affect the types of enzymes produced. N additions can also directly inhibit the production of some enzymes secreted by soil fungi^24^.

### Responses of soil ammonia-oxidizing bacteria and archaea to nitrogen applications

Increased N additions resulted in increased rates of net nitrification. Similar results were found in other semi-arid grassland soils^28^,^30^. The results also showed the increase of soil NO_3_^−^-N and no accumulation of NH_4_^+^-N concentration in 0–112 kg N ha^−1 ^yr^−1^ treatments, as the NH_4_^+^-N converted from urea was rapidly oxidized by ammonium oxidizer to NO_3_^−^-N in the grassland soil. It is noteworthy that soil NH_4_^+^-N significantly accumulated in 392 kg N ha^−1 ^yr^−1^ treatment despite the net nitrification rate increased, indicating that the amount of urea addition had remarkably exceeded the substrate demand of ammonium oxidizer. We found that the soil NO_3_^−^-N content decreased in 224 and 392 kg N ha^−1 ^yr^−1^ treatments, which was inconsistent with the trend of soil net nitrification rate in these treatments. It could be interpreted that high nitrification potential promoted denitrification process and converted more NO_3_^−^-N to nitrite, subsequently resulting in N_2_O or N_2_ emissions.

Zhang *et al*.^28^ found that soil microbial biomass carbon and soil microbial activity of bacterial communities tended to increase at low levels of urea additions; they peaked when the N addition rate was 160 kg N ha^−1 ^yr^−1^, and then decreased under the 320 kg N ha^−1 ^yr^−1^ treatment. The AOB patterns in our study were similar to the microbial activity of the bacterial community reported by Zhang *et al*.^28^. Concentrations of NH_4_^+^-N were relatively low and corresponded to the increased trends of NO_3_^−^-N concentrations and nitrification rates in the 56–112 kg N ha^−1 ^yr^−1^ treatments in our study, indicating that active nitrification was induced by soil ammonia oxidizing prokaryotes. The advantage of AOA over AOB in abundance in N_0_ was gradually transformed into the quantitative superiority of AOB over AOA under urea additions, especially with high urea additions (N_224_, N_392_), suggesting that AOB was more acclimatized to urea additions than AOA in the semi-arid grassland. This was likely because that the NH_4_^−^-N content increased with urea additions which decreased the abundance of AOA, increasing the abundance of AOB.

This finding was in agreement with the results of Jia and Conrad^31^ and Xia *et al*.^32^ who found that soil AOB dominated ammonia oxidation at fertilizer application of 160 kg N ha^−1 ^yr^−1^. Wang *et al*.^30^ found that urea application of 180 kg N ha^−1 ^yr^−1^ increased AOB abundance in acidic soils, and it was further confirmed by the positive relationships between AOB and NO_3_^−^-N in our study. AOB, rather than AOA, promote nitrification when ammonia concentrations are elevated^33^. The similar trend of AOA and AOB showed in our experiment was also reported in a no P addition study conducted by Shen *et al*.^13^.

The results also showed that AOA remained steady under urea applications of 0–224 kg N ha^−1 ^yr^−1^ but decreased significantly for application of 392 kg N ha^−1 ^yr^−1^. Similarly, Shen *et al*.^13^ found that urea applications of 0–320 kg N ha^−1 ^yr^−1^ did not have any influence on AOA, but noticed a significant decrease in AOA when 640 kg N ha^−1 ^yr^−1^ urea was applied to semi-arid grassland. This may indicate that AOA can adapt to relatively low N availability environment^30^ and that they can grow in a broad range of N-supply environments^14^,^33^,^34^. Additionally, AOA is an important driven factor for nitrification in low-nutrient containing environment^33^, and AOB is positively responded in nitrogen-rich soils^14^. Furthermore, Gubry-Rangin *et al*.^35^ found that AOA abundance increased with soil pH. We also found a positive relationship between AOA and soil pH, which suggests that soil acidity might cause AOA to decrease under the highest application of N (392 kg N ha^−1 ^yr^−1^); this is consistent with previous studies^36^,^37^ which reported that different AOA phylotypes were selected by different soil pH.

In summary, we found that LAP and NAG activities were significantly inhibited by urea applications of 224 and 392 kg N ha^−1 ^yr^−1^ over a seven-year period, respectively, in semi-arid temperate grasslands. Correlation analysis showed that there was a negative relationship between LAP and NAG activities and soil NH_4_^+^-N content, indicating that ammonia had an inhibitory effect on N hydrolase. The abundance of AOB increased when urea additions were between 56–224 kg N ha^−1 ^yr^−1^. There was a significant positive relationship between the NO_3_^−^–N content and AOB abundance. The abundance of AOA was not sensitive to urea additions of 56–224 kg N ha^−1 ^yr^−1^. The results indicate that N additions between 56 and 224 kg N ha^−1 ^yr^−1^ would be beneficial and 224 kg N ha^−1 ^yr^−1^ can be a threshold for AOB abundance in this semi-arid grassland in Inner Mogolia.

## Methods

### Study Site

The seven-year field experiment was conducted in a typical temperate grassland in Xilinguole, in the Inner Mongolia Grassland Ecosystem Research Station (IMGERS), China (43°38′ N, 116°42′ E) commencing in 2006. The mean annual precipitation was 345 mm and the mean annual temperature was 1.1 °C (1980 to 2010 data)^38^. July is the warmest month, with mean temperature of about 19 °C^3^. The soil is classified as dark chestnut (Calcic Chernozem according to ISSS Working Group RB 1998) and the dominant plant species in the region are perennial rhizomatous grasses (*Leymuschinensis*, *Stipagrandis*, *Cleistogenessquarros*a and *Agropyronmichnoi*).

### Experimental design

This N rate field trial had a completely randomized plot design and was initiated in grassland dominated by *Leymuschinensis* in 2006. The field had been fenced since 1999 to prevent grazing by large animals. Detailed information of the experimental design used in this research has been reported previously^39^,^40^. We used 5 rates of urea-N with 3 replicates, giving a total of 15 experimental plots (6 m × 6 m) with a 1 m walkway between each plot. The N treatments were 0, 56, 112, 224, and 392 kg N ha^−1 ^yr^−1^ (designated N_0_, N_56_, N_112_, N_224_, and N_392_). The fertilizer was thoroughly mixed with sand and then was applied to the plot surfaces in late May each year from 2006 to 2012. According to Lü and Tian^41^, N deposition in the experimental area is approximately 4 kg ha^−1 ^yr^−1^. In order to simulate the effects of long term N deposition in relatively short term experimental period, and to assess the effects of N fertilization on enzymatic and microbial processes affecting soil N transformations, N application rates were 10 to 100 times higher than the background atmospheric N deposition in this study site. Each plot also received 15.5 kg P ha^−1 ^yr^−1^ in the form of KH_2_PO_4_, to prevent P limitation^1^,^3^.

### Soil sampling

Soil samples were collected from the 0–20 cm layer in July 2012. Five soil cores (5 cm in diameter) were taken from each plot and then mixed to form a composite sample. The soil samples were kept in polyethylene bags, and were placed in a container with ice. On arrival at the laboratory, soil samples were sieved through a 2 mm mesh. Approximately 2 g of each soil sample was placed in an autoclaved microcentrifuge tube (2 mL) and stored at −80 °C for DNA extraction, while another soil sub-sample was stored at 4 °C for the determination of enzyme activity, net nitrification rate, and NH_4_^+^-N and NO_3_^−^-N concentrations. The remaining soil samples were air-dried and used for determination of soil pH and total organic carbon (SOC).

### Soil property analysis

The net nitrification rate was determined using a laboratory incubation procedure^42^. We collected soil samples in July and analyze the net nitrification rate at the similar temperature as July. Samples (10 g) of field moist soil were incubated in 100 ml polyethylene bottles at 20 °C in the dark for 14 days. The net nitrification rate on a dry mass basis was calculated as the change in NO_3_^−^-N between the initial samples and the incubated samples (mg NO_3_-N kg^−1^ soil d^−1^). Total soil organic carbon (SOC) was determined by a CN Analyzer (Vario Max, Elementar, Germany) using a high temperature combustion method. Soil NH_4_^+^-N and NO_3_^−^-N were analyzed from filtered 2M KCl-extracts colorimetrically with a continuous flow analyzer (AutoAnalyzer3, BranLuebbe, Germany). Soil pH was measured with a glass electrode in a 1:2.5 soil/water suspension^43^.

### Enzyme activity analysis

The activities of LAP and NAG were measured using the method of Saiya-Cork *et al*.^24^. L-Leucine-7-amino-4-methylcoumarin and 4-MUB-N-acetyl-b-D-glucosaminide (each at 250 μM) were used as substrates for LAP and NAG, respectively. The microplates were incubated in the dark at 20 °C for up to 4 h. Fluorescence was measured using a microplate fluorometer (Synergy^H4^, BioTek) with 365 nm excitation and 450 nm emission filters. Soil enzyme activities were calculated using the method of German *et al*.^44^ and were expressed in units of nmol g^−1 ^h^−1^.

### Soil DNA extraction and quantitative PCR of amoA genes

DNA was extracted from the three replicate plots from each N addition treatment. Soil DNA was extracted from 0.5 g of fresh soil using the Fast DNASPIN Kit for Soil (Q BIO gene Inc., Carlsbad, CA, USA), following the manufacturer’s protocol. The extracted DNA was diluted in 75 μL of sterile deionized water and stored at −20 °C until analysis. Quantitative PCRs of archaea and bacterial *amoA* genes were amplified with the primers *Arch-amoA*F/*Arch-amoA*R^45^ and *amoA1*F/*amoA2*R^46^, respectively. To prepare the standardization templates, the purified *amoA* gene PCR product was cloned into the pMD18-T plasmid vector (TaKaRa, Dalian, China) and transformed into competent *Escherichia coli DH5α* cells, following which the plasmid was extracted using a Wizard Plus SV Minipreps DNA Purification System (Promega, USA). The DNA concentration was determined using a NanoDrop ND-2000 UV-VIS spectrophotometer (Thermo Scientific). The standard DNA curve template was the plasmid DNA diluted with sterile deionized water from 10^−1^ to 10^−8^. Gene amplifications of AOA and AOB *amoA* were done on a Light Cycler 480 (Roche, Swiss) and an iCycleriQ 5 thermocycler (Bio-Rad), respectively. The coefficient of determination (*r*^2^) and the amplification efficiency for the amplification standard curve of AOA *amoA* gene were 0.997 and 87.4%, respectively, while the *r*^2^ and the amplification efficiency for the AOB *amoA* gene were 0.991 and 85.7%, respectively. Amplification programs are described by He *et al*.^36^.

### Statistical analysis

The amoA gene copy numbers were log-transformed so they corresponded with a normal distribution. Statistical analyses were performed using SPSS 17. One-way analysis of variance (ANOVA) and Duncan’s multiple comparisons were used to determine the influence of N treatments on soil properties using a significance level of *P* <  0.05. Linear and cubic regression analyses were used to explore the effect of nitrogen additions on soil N hydrolytic enzyme activity and ammonia-oxidizing microbial abundance. Pearson correlation analysis was used to explore the relationships between soil nutrient contents, soil enzyme activities and amoA gene copy numbers.

## Additional Information

**How to cite this article**: Zhang, X. *et al*. Responses of soil hydrolytic enzymes, ammonia-oxidizing bacteria and archaea to nitrogen applications in a temperate grassland in Inner Mongolia. *Sci. Rep.* **6**, 32791; doi: 10.1038/srep32791 (2016).

## Figures and Tables

**Table 1:** Correlations between soil physico-chemical properties, soil hydrolytic enzyme activities and ammonia-oxidizing bacteria and archaea under N fertilization practices in 2012.

	pH	Nitrification (mg NO_3_-N kg soil h^−1^)	SOC (g kg^−1^)	NH_4_-N (mg kg^−1^)	NO_3_-N (mg kg^−1^)
NAG (nmol g^−1^ soil h^−1^)	**0**.62^*^	−**0**.75^**^	−**0**.59^*^	−**0**.69^*^	−0.32
LAP (nmol g^−1^ soil h^−1^)	**0**.65^**^	−**0**.80^**^	−**0**.57^*^	−**0**.83^**^	−0.09
AOA (Log number of gene copies g^−1^ soil)	**0**.54^*^	−**0**.77^**^	−**0**.53^*^	−**0**.91^**^	0.16
AOB (Log number of gene copies g^−1^ soil)	−0.36	0.06	0.42	−0.10	**0**.66^*^

Note: The values are correlation coefficients. *P < 0.05, **P < 0.01; NAG, *β*-1,4-N-acetylglucosaminidase; LAP, L-leucine aminopeptidase; AOA, ammonia-oxidizing archaea; AOB, ammonia-oxidizing bacteria; SOC, soil organic carbon content.
